# Enhanced tumor control activities of anti-mPD-L1 antibody and antigen-presenting cell-like natural killer cell in an allograft model

**DOI:** 10.1186/s12885-024-11889-4

**Published:** 2024-01-26

**Authors:** Yi-Ping Hung, Chia-Chun Tu, Jiun-I Lai, Muh-Hwa Yang, Jan-Mou Lee, Yee Chao

**Affiliations:** 1 Division of Medical Oncology, Department of Oncology, Taipei Veterans General Hospital, Taipei, Taiwan; 2 Institute of Clinical Medicine, School of Medicine, National Yang Ming Chiao Tung University, Taipei, Taiwan; 3 School of Medicine, National Yang Ming Chiao Tung University, Taipei, Taiwan; 4 FullHope Biomedical Co., Ltd, New Taipei City, 241405 Taiwan; 5 Center of Immuno-Oncology, Department of Oncology, Taipei Veterans General Hospital, Taipei, Taiwan; 6 Department of Medicine, Central Clinic and Hospital, Taipei, 106441 Taiwan

**Keywords:** Adoptive cell therapy, Immune checkpoint inhibitor, Immunotherapy, NKDC, Tumor microenvironment

## Abstract

**Background:**

Despite the utilization of immune checkpoint inhibitors (ICIs) in treating numerous types of cancers being approved, their efficacy in tumor control in the clinic is not satisfactory. Since adoptive cell therapy (ACT) can alter the tumor microenvironment, we hypothesized that ACT potentially synergized with ICI in tumor control and examined this hypothesis via a murine allograft model.

**Methods:**

Female C57BL/6 mice were stimulated with interleukin 15 and granulocyte monocyte-colony stimulating factor, followed by collecting their bone marrow cells for murine NKDC cultivation. Then, female C57BL/6 mice, inoculated with lymphoma cancer cell line E.G7-OVA, were administrated with murine NKDC cells, murine anti-program cell death ligand-1 antibody (α-mPD-L1), or both for 28 days. After 28 days of treatment, mice were sacrificed whose inoculated tumors, spleen, sentinel lymph nodes, and peripheral blood were collected to measure tumor size, lymphocyte infiltration, and change of immune cell profile.

**Results:**

Combined treatment of NKDCs with α-mPD-L1 exhibited significantly stronger tumor control efficacy than treatment of NKDCs or α-mPD-L1 alone. NKDCs/α-mPD-L1 combination increased migration of dendritic cells, CD4, CD8 T cells, and activated CD8 T cells to the tumor-bedding site, and promoted endogenous tumor-specific cytotoxic T-cell response.

**Conclusion:**

The current study confirmed our hypothesis that combining NKDC ACT with ICI therapy can potentiate tumor control efficacy by manipulating the tumor microenvironment. This study provided a novel circumstance on tumor immunotherapy.

**Supplementary Information:**

The online version contains supplementary material available at 10.1186/s12885-024-11889-4.

## Introduction

Immune surveillance of tumor cells refers to the destruction of tumor cells by natural killer cells (NKs) and the presentation of tumor antigens to the adaptive immune system (T-cell response and humoral response) by antigen-presenting cells (APCs, such as dendritic cells [DCs]) [1, 2]. However, some tumor cells form an immunosuppressive tumor microenvironment (TME) to avoid attacks from the immune system, which includes downregulation of tumor antigens, upregulation of inhibitory receptors (e.g., programmed cell death protein 1 [PD-1], and programmed cell death protein ligand 1 [PD-L1]), and stimulation of regulatory cells accumulation [3]. Therefore, cancer immunotherapy intends to ameliorate immunosuppressive TME through breaking PD-1/PD-L1 interaction (by immune checkpoint inhibitors [ICIs]) and enhancing tumor-specific immune response (for example, via adoptive cell therapy [ACT]) [4].

Although the regulatory bodies around the world approved the application of ICI immunotherapies in both solid and liquid tumors, the clinical response of ICI immunotherapies is still challenging owing to impaired tumor-antigen-presentation and tumor-specific killing [5, 6]. To overcome the limitation of ICI immunotherapies, combination ICIs with ACTs, either cytotoxic cell-based or antigen-presenting cell-based, is proposed [7]. However, cytotoxic-cell-based ACTs can only deal with tumor carrying known and conserved antigens [8]. Even though antigen-presenting cells-based ACTs can promote tumor control activity via orchestrating antigen-specific adaptive immune response without ex vivo education, lack of expression of the major-histocompatibility complex class I upon solid tumors leads tumor cells evading from the antigen-presentation [9]. Previous studies revealed that some natural-killer cell (NK) subpopulations acted as antigen-presenting cells during infection [10]. Also, we discovered a novel NK subpopulation, antigen-presenting cell-like NKs (NKDCs), which carried the phenotypes and displayed both activities of NKs and DCs. The current study intended to evaluate whether NKDC can synergize with ICIs in tumor control. In the study, we constructed an allograft murine tumor model which included murine lymphoma cell line E.G7-OVA (E.7, ELF4 lymphoma cell line transfected with ovalbumin) as the target, murine NKDCs cultured from bone marrow cells (BMs) and anti-murine PD-L1 antibody (α-mPD-L1) as effectors.

## Methods & materials

### Reagents and antibodies

Reagents and antibodies applied in this study were enlisted in Supplementary Tables 1 and 2. All reagents and antibodies were aliquoted as received and stored under recommended condition from manufacture until used.

### Cell line

We obtained E.7 cell line (CVCL_3505) from Bioresource Collection and Research Center (BCRC, Hsinchu, Taiwan) and maintained cells in the recommended medium from the vendor and renewed once 2–3 days. All experiments were performed within ten passages to keep uniformity.

### Animals

We obtained C57BL/6 mice (female, 6–10 weeks old, *n* = 78) and BALB/c mice (female, 4–6 weeks old, *n* = 6) from National Laboratory Animal Center (Taipei, Taiwan), fed the mice in controlled conditions (21 ± 2^o^C, 55 ± 10% humidity, 12/12 h day/night cycle) and *ad libitum* food and water from the specific pathogen-free animal facility of Fu-Jen University laboratory animal center. The designation of the animal experiment complied with ARRIVE guidelines and the “Guide for the Care and Use of Laboratory Animals” from the National Research Council, and the study protocol was reviewed and approved by The Institutional Animal Care and Use Committees of Fu-Jen University (approval code P10824-修1).

### Immunostaining and flow cytometry

The immunostaining and flow cytometry process followed the description in our previous study [11]. We used Kaluza analysis software (V2.1, Beckman-Coulter, Brea, CA, USA) in the data collection of flow cytometry.

### Maintenance and functional evaluation of murine NKDC

#### NKDC preparation and phenotypic analysis

Murine NKDC was cultured from the murine BMs collected from sixty C57BL/6 mice [12]. Collected BMs were cultured (1 × 10^6^ cells/mL) with complete medium, comprising Roswell Park Memorial Institute medium 1640, fetal bovine serum (10% v/v), murine interleukin 15 (12.5 ng/mL), and murine granulocyte-macrophage colony-stimulating factor (5 ng/mL, applied on days 3 and 6), for nine days. NKDC was collected through centrifugation and utilized in the following analysis. For phenotype determination, NKDCs were incubated with an Fc blocker followed by immunostaining and analysis by the flow cytometer (Navios, Beckman-Coulter). Identification of NKDCs followed a gating pedigree described in Supplementary Fig. 1.

#### Cytotoxicity assay

The killing activity of NKDC was determined by the PanToxilux kit (OncoImmunin, Inc., Gaithersburg, MD, USA), which determination followed the protocol in the manual. In brief, E.7 cells were stained with TFL4 (viable dye) and incubated with NKDC (or cultured medium; cell ratio: E.7: NKDC = 1: 20) for 50 min. The cultured cells were subsequently stained with PS (fluorescent-labeled caspase substrate) and analyzed fluorescence by flow cytometry. We defined the PS fluorescence from the group without NKDC co-incubation as non-apoptotic cells, and the cells carrying stronger PS fluorescence than non-apoptotic cells were apoptotic cells.

### Mixed lymphocyte reaction (MLR)

The antigen-presenting activity of NKDCs was assessed by MLR [13]. Briefly, lymphocytes were isolated from axillary lymph nodes of BALB/c mice [14]. Isolated lymphocytes were stained with CellTrace® Violet (CTV; Thermo-Fisher, Waltham, MA, USA) and co-cultured with NKDCs (or complete medium) for five days. After cultivation, cells were applied for CTV-fluorescence analysis by flow cytometry. We defined the CTV fluorescence from the group without NKDC co-cultivation as non-proliferated cells, and the cells carrying weaker CTV fluorescence than non-proliferated cells were proliferated cells.

### In vivo evaluation of tumor-control of NKDC/mPD-L1

#### Construction of E.7 allograft model and NKDC administration

**Fig. 1:**
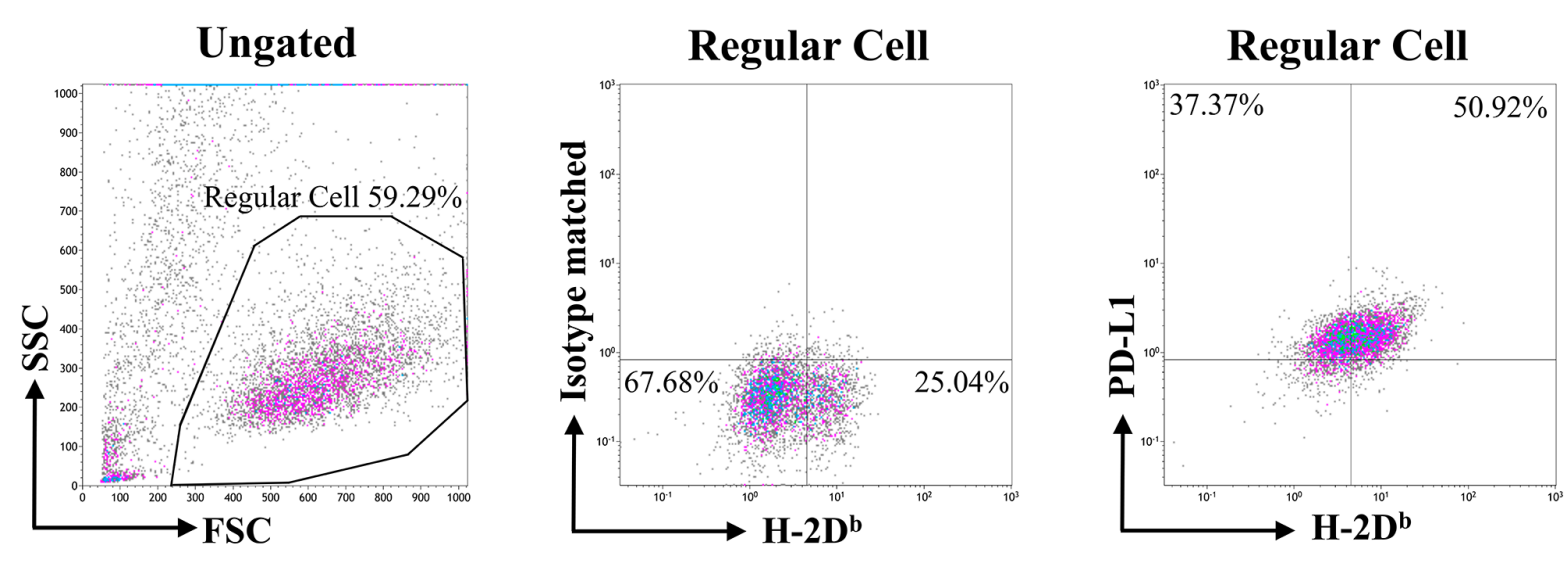
Schematic illustration of allograft E.7 tumor model Six-week-old of female C57BL/6 mice were randomly assigned into 5 groups: 1× PBS, E.7, E.7 + anti-PD-L1 antibody (α-mPD-L1), E.7 + NKDC, and E.7 + α-mPD-L1 + NKDC, and administrated with α-mPD-L1, NKDCs, both, or PBS, respectively, for 28 days

The detailed administrating schedule of the allograft tumor model is available in Fig. 1. In brief, eighteen C57BL/6 mice were randomly assigned into five groups and utilized in study treatment regarding their study grouping on Day 0. On the same day, mice were inoculated with 5 × 10^5^ cells/mice of E.7 cells or PBS. Later, intraperitoneal injection of α-mPD-L1 (6.25 mg/kg body weight) was performed on Days 3, 10, and 17. Intravenous inoculation of NKDCs (1 $\times$ 10^7^ cells/mice) was initiated on Day 7 and repeated once on Day 15. We weighed the body weight and tumor volume every 2–3 days during the treatment [15]. On Day 28, mice were sacrificed via CO_2_ anesthetization, and peripheral blood, tumor, spleen, and sentinel lymph nodes from each mouse were collected in terms of the study group for preparing single-cell suspensions.

#### Preparation of single-cell suspension

Single-cell suspensions of tumors, spleens, and sentinel lymph nodes were prepared via homogenization [16]. Erythrocytes in the peripheral blood sample were removed via ACK lysis [16]. All cell suspensions were stored under 4^o^C until use.

#### Tumor-specific T-cell-activation assay

Evaluating the re-stimulation of E.7-specific T cells followed the protocol described in the literature with modification [17]. Mice splenocytes (without T-cell isolation) were stained with CTV followed by cultivation with OVA peptide for five days. Cell stimulation cocktails were applied at 2 h before cell harvesting.

### Statistical analysis

The result of each experiment was summarized from at least three repeats and plotted by Prism V6 (GraphPad Inc., La Jolla, CA USA). We utilized Prism in determining statistical significance among each comparison which unpaired Student-t test (for pairwise comparison) and one-way ANOVA plus Tukey’s test (for multiple comparisons) were utilized, and α value was 0.05.

## Results

### NKDC exhibited both NK and DC activities

**Fig. 2:**
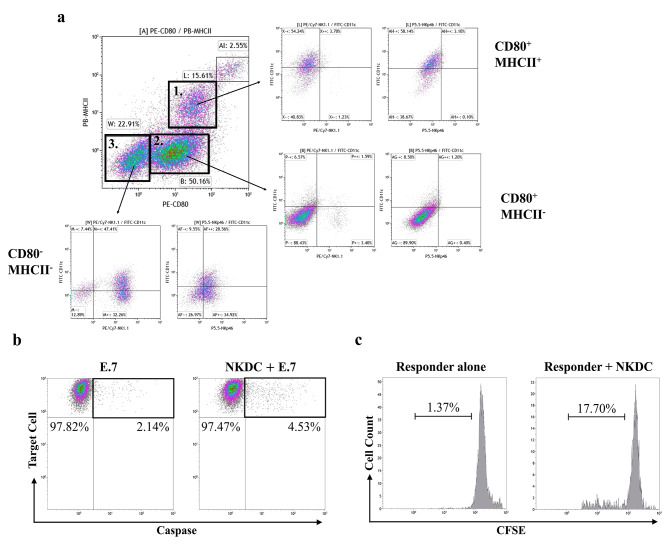
NKDC displayed both natural killer cells and dendritic cells activity Antigen-presenting cell-like natural killer cells (NKDCs) were generated from murine bone marrow cells by culturing them with interleukin 15 and granulocyte-macrophage colony-stimulating factor for 9 days. After cultivation, (**a**) Phenotypes, (**b**) cytotoxicity against E.G7-OVA cells (E.7 cells), and (**c**) antigen-presenting activity were evaluated. Ratios among NKDCs, E.7 cells and BALB/c lymphocytes were 1:20 and 5:1, respectively

We showed the phenotype of NKDCs in Fig. 2A. NKDC comprised three predominant populations: CD80^+^MHCII^+^CD11c^+^NK1.1^-^NKp46^-^ (population 1), CD80^+^MHCII^-^CD11c^-^NK1.1^-^NKp46^-^ (population 2), and CD80^-^MHCII^-^CD11c^-^NK1.1^+^NKp46^+^ (population 3), respectively. As co-incubating NKDCs with TFL-4-labeled E.7 cells with a ratio of 20:1, the percentage of apoptotic E.7 cells increased from 2.14 to 4.53% (Fig. 2B). A similar result was observed in the MLR assay, in which NKDC triggered CFSE-labeled BALB/c lymphocyte proliferation in a ratio of 5:1 (Fig. 2C). These data indicated that NKDCs displayed the activities of NK and DC.

### NKDCs showed additive enhancement in tumor control while treating alongside α-mPD-L1

**Fig. 3:**
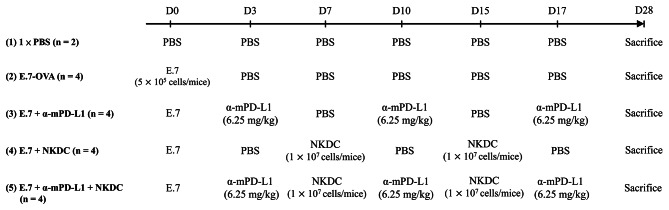
E.7cells expressed abnormal MHCI and significant PD-L1 E.7 cell was stained with antibodies targeting to H-2D^b^ (a subunit of C57BL/6 MHCI) and PD-L1 followed by fluorescent analysis by flow cytometry

**Fig. 4:**
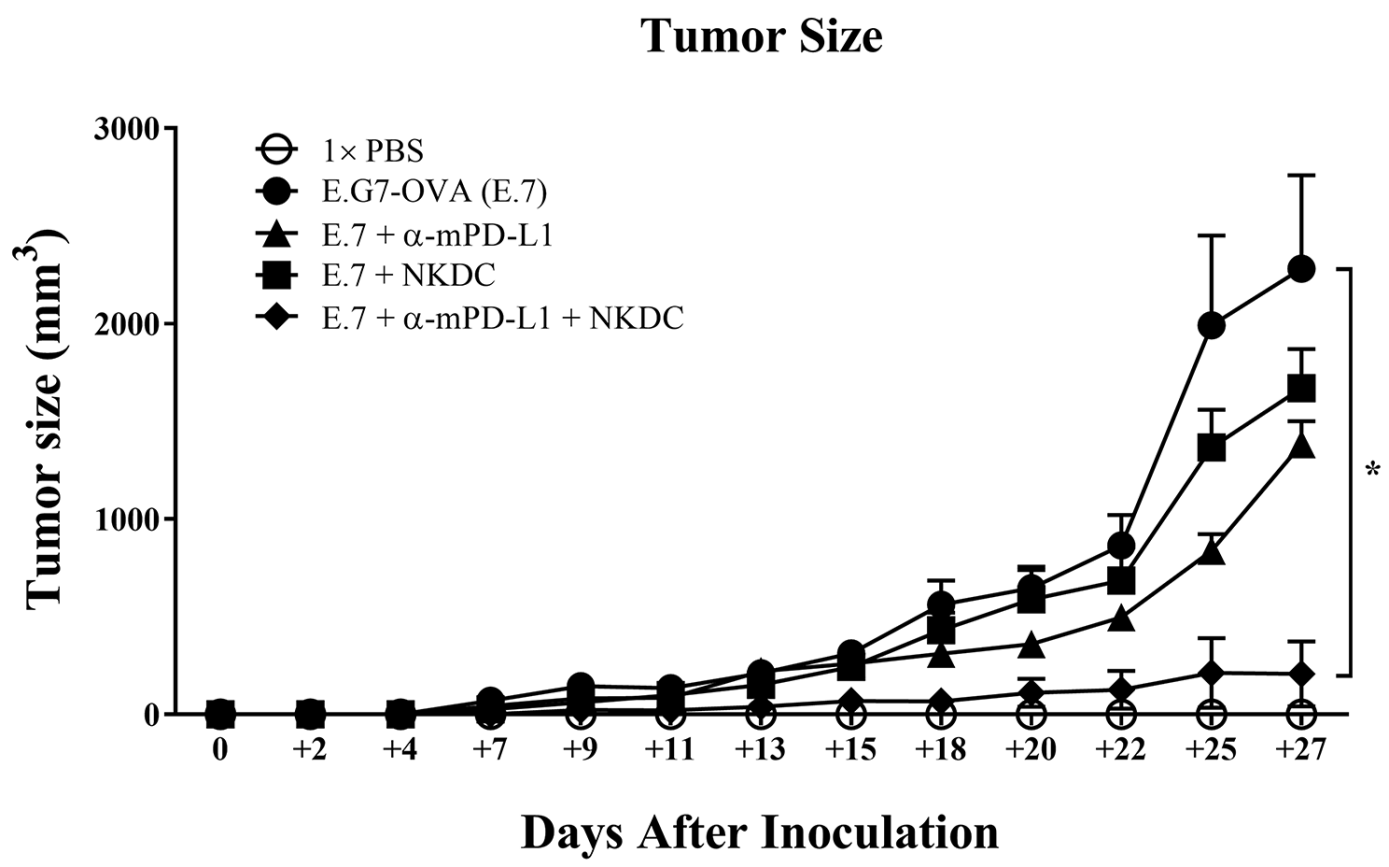
Adoptively transferred NKDC showed synergetic effects with α-mPD-L1 antibody in E.7allograft mouse model A total of 18 Female C57BL/6 mice were randomly grouped into five, followed by E.7 cell inoculation. Administration of NKDC and α-mPD-L1 followed the treatment schedule described in Fig. 3, respectively. The tumor volume of each mouse was measured every 2–3 days until sacrifice and showed in a line plot with mean ± SEM. *, *p* < 0.05

To evaluate whether NKDC/ICI combination enhanced the efficacy of tumor control, we constructed E.7-based allograft tumor model and treated the allograft mice with NKDC, ICI, and both for 28 days (Fig. 3). In the model, E.7 cells were chosen as target cells based on the positive expression of PD-L1 on the cells (Fig. 1). During the treatment, the body weight of each group was comparable with 1× PBS, indicating no acute toxicity occurred in the treatment (**Data not shown**). Tumor volumes among E.7, E.7 + α-mPD-L1, and E.7 + NKDC groups were comparable at Day 28 (all *p* > 0.05, Fig. 4). Notably, tumor volume of E.7 + α-mPD-L1 + NKDC group was significantly smaller than that of E.7 group and two mono-treatment groups at Day 28 (*p* < 0.01, Fig. 4). This result revealed the observable enhancement in tumor control as NKDCs combined with α-mPD-L1.

### α-mPD-L1/NKDC combination increased the amount of DCs in the tumor-bedding site

**Fig. 5:**
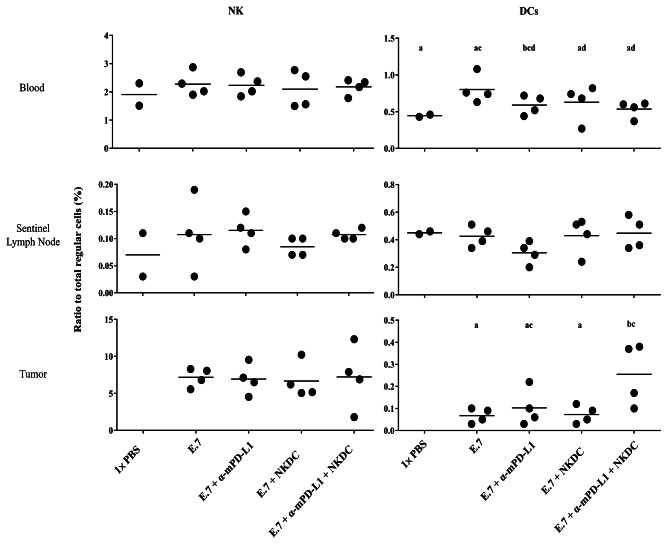
α-mPD-L1 and NKDC triggered DCs accumulation in tumor-bedding site NKs and DCs in single-cell suspensions of peripheral blood, sentinel lymph nodes, and tumor-bedding sites were identified via pedigree described in Supplementary Fig. 2. Means not sharing ant letters were significantly different by the Tukey’s test with 5% level of significance

To evaluate how α-mPD-L1 promote tumor control activity of NKDC, amounts of NKs and DCs in the single-cell suspensions from peripheral blood, sentinel lymph nodes, and tumor-bedding sites were determined (Supplementary Fig. 2). As shown in Fig. 5, the proportion of NKs in peripheral blood, sentinel lymph nodes, and tumor-bedding sites among each group was comparable. Of note, a significantly higher proportion of DCs in the tumor-bedding site was observed in E.7 + α-mPD-L1 + NKDC group compared with other groups (*p* < 0.05), revealing a promotion of the accumulation of DCs in the tumor-bedding site may cause tumoricidal promotion of α-mPD-L1/NKDC combination.

### α-mPD-L1/NKDC combination promoted amounts of myeloid-derived suppressor cells (MDSCs) in sentinel lymph nodes

**Fig. 6:**
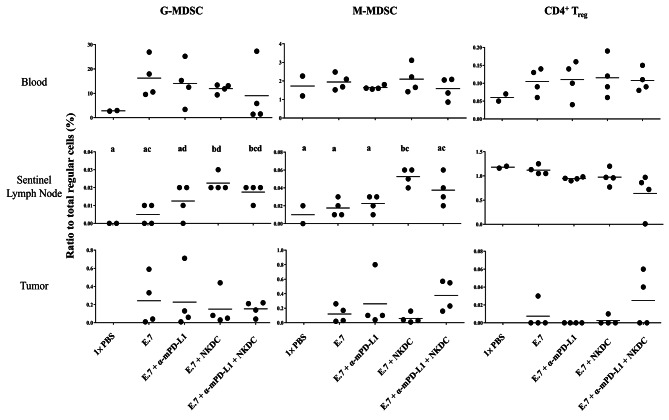
NKDC/α-mPD-L1 synergism increased amount of granulocytic myeloid-derived suppressor cells (G-MDSCs) in sentinel lymph nodes Granulocytic-myeloid-derived suppressor cells (G-MDSC), monocytic-MDSCs (M-MDSC), and regulatory CD4^+^ T cells (CD4^+^ T_reg_ cells) within single-cell suspensions of peripheral blood, sentinel lymph nodes, and tumor-bedding sites were identified via pedigree described in Supplementary Fig. 3. Means not sharing ant letters were significantly different by the Tukey’s test with 5% level of significance

Combination of α-mPD-L1 and NKDC may contribute to manipulating the distribution of regulatory cells [18]. Hence, we further investigated the distribution of G-/M-MDSC and CD4^+^ T_reg_ cells (Supplementary Fig. 3). Significant increases of MDSCs in sentinel lymph nodes were observed in either the NKDC monotherapy group or combination therapy group when compared with the untreated group (both *p* < 0.05), which were not observed in the peripheral blood and tumor-bedding site (Fig. 6). Comparable amounts of CD4^+^ T_reg_ cells in all single-cell suspensions were observed among all groups. This result showed that α-mPD-L1/NKDC combination did not alter regulatory cell distribution.

### α-mPD-L1/NKDC combination promoted activation of CD8 T cells in tumor-bedding sites

**Fig. 7:**
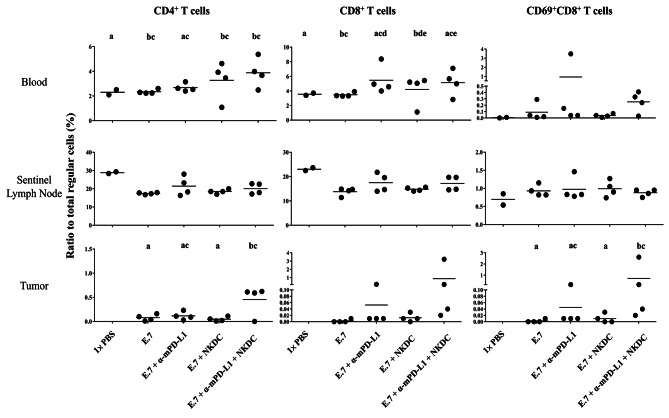
NKDC/α-mPD-L1 synergism promoted CD4^+^ T and activated CD8^+^ T cells accumulation in tumor-bedding sites CD4^+^, CD8^+^, and activated CD8^+^ (CD69^+^CD8^+^) T cells in single-cell suspensions of peripheral blood, sentinel lymph nodes, and tumor-bedding sites were identified via pedigree described in Supplementary Fig. 4. Means not sharing ant letters were significantly different by the Tukey’s test with 5% level of significance

Increased DC amounts in tumor-bedding sites potentially enhanced tumor-specific T-cell response [19]. Therefore, we measured the distribution of CD4^+^ T, CD8^+^ T, and activated CD8^+^ T cells (CD69^+^CD8^+^) in peripheral blood, sentinel lymph nodes, and tumor-bedding sites (Supplementary Fig. 4). Significantly higher amounts of CD4^+^, CD8^+^, and activated CD8^+^ T cells within the tumor-bedding site were observed in the E.7 + α-mPD-L1 + NKDC group compared with those in the mono-treated and un-treated groups (all *p* < 0.05; Fig. 7), indicating DC-associated accumulation and activation of T cells may contribute to enhance tumor control of α-mPD-L1/NKDC combination.

### α-mPD-L1/NKDC combination reinforced cognate tumor antigen-specific cytotoxic T cell responses

**Fig. 8:**
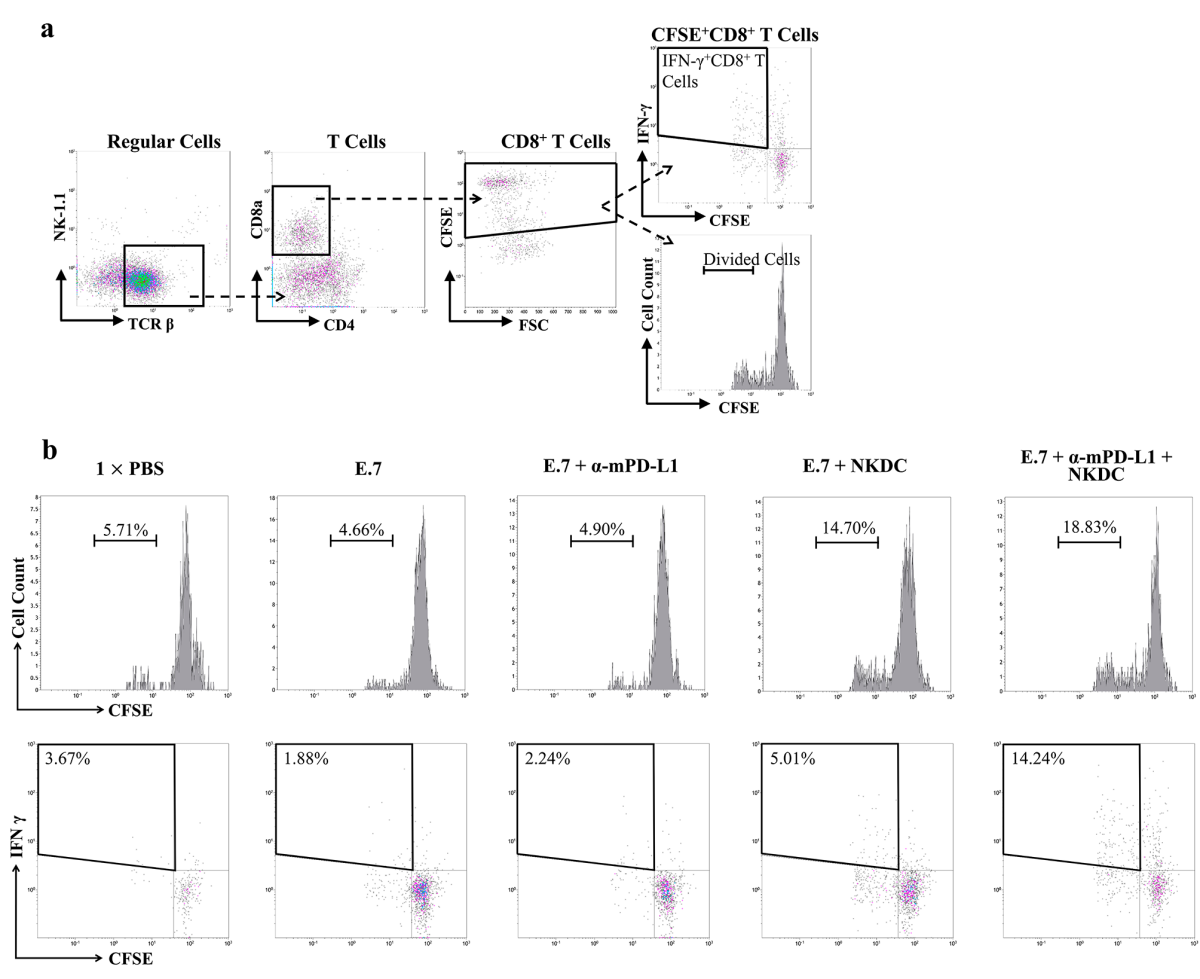
Adoptively transferred NKDC synergized with α-mPD-L1 antibody to mount more efficient cognate tumor antigen-specific cytotoxic T cell responses (**a**) Total cultured CFSE-labeled cells were harvested on day 5, and the CFSE diluted patterns on CD8^+^ T cells were determined. (**b**) Each histogram and bi-axis-dot plot represents the data out of 2 (1× PBS group) to 4

The T cells of the recipients’ spleen were CFSE labeled and stimulated with OVA peptide to investigate the sustained tumor-specific immune activation in recipient mice. As the results showed in Fig. 8A, E.7 + NKDC (14.7%) and E.7 + combination therapy groups (18.83%) exhibited a significantly higher proportion of proliferated T cells than in the E.7 + α-mPD-L1 group (4.9%, *p* = 0.0008; Fig. 8B), indicating that NKDC alone and α-mPD-L1 strengthened tumor-specific T-cell memory. In addition, IFN-γ expression was higher in the E.7 + α-mPD-L1 + NKDC combination group than in the other groups (14.24%, *p* = 0.0113; Fig. 8B). Our results indicate that combined treatments of NKDCs and α-mPD-L1 lead to sustained tumor-specific immune activation that correlates with tumor control activity in the recipient mice models.

## Discussion

The current study described an additive enhancement of tumor control between α-mPD-L1 and NKDCs and its detailed mechanism via the murine allograft tumor model. The combination of NKDCs and α-mPD-L1 exhibited an enhanced efficacy of tumor control than NKDC and α-mPD-L1 alone. α-mPD-L1/NKDC combination increased DC and T cell accumulation in the tumor-bedding site and such increase of DC and T cell enhanced intensity and sustained tumor-specific T-cell response.

ICI immunotherapies break immune tolerance toward tumor cells via reducing PD-1/PD-L1 interaction and subsequently initiate endogenous antitumor immune response [20]. For those cancer patients experiencing immune dysregulation, the antitumor immune response cannot be well-developed owing to compromised peripheral immunity [20]. Hence, combinations of ICI immunotherapies with other cancer treatments (e.g., target therapies, ACTs of tumor-specific immune cells, innate immune agonists, or anti-regulatory cell therapies) are suggested to ameliorate the compromised peripheral immunity in cancer patients [21]. Hui et al. reported a case that administrated autologous cytokine-induced killer cells (CIK, immune cell product mixed with NKs and cytotoxic T cells) and pembrolizumab to a patient with metastatic squamous cell carcinoma in the lung [22]. They observed a rapid decrease in tumor volume and serum tumor markers after treatment, indicating that combining ACT and ICIs treatments in improving tumor control was feasible [23]. In the report from Zhou et al., the disease-control rate (DCR) of non-small-cell lung cancer patients treating with CIK and sintilimab (anti-PD-1 antibody drug) was 64.7% [24]. Furthermore, four clinical studies revealed a highly 12-week DCR (61.5–100%) observed in patients with solid tumors after treatment of autologous tumor-infiltrated lymphocytes and ICIs [7, 25–27]. These studies demonstrate that ICI plus adoptive transferring of tumor-specific immune cells can ameliorate compromised peripheral immunity of cancer patients and lead to better tumor control than ICI or ACT alone. We identified that NKDCs carry both NK and DC activity (Fig. 2B & C), which can do tumor-cell killing and antigen-presentation of tumor-specific antigens to T cells and trigger an adaptive immune response from one cell product. This property implied that antigen-presenting and tumor-specific killing activities of NKDCs can be well-functioned in tumors carrying unknown or heterogenic tumor antigens, which frequently obstacles the application of T-cell-based and NK-based ACTs [8, 28].

Tumor cells express PD-L1 to reduce tumor-mediated T-cell and NK activation [29, 30]. Blocking PD-L1 activity can stimulate the endogenous tumor-specific immune response. Recent studies discovered that DC also expresses PD-L1 and that expression of DCs controls stimulatory activity toward T cells [31]. PD-L1 interacts with PD-L1 in *cis* or *trans* manners and triggers CD28-related signaling on T cells and consequently activated T cells [32, 33]. While PD-L1 expression is more abundant than CD80, PD-1/PD-L1 signaling will offset CD28-related signaling [33]. Therefore, blocking PD-L1 can either activate endogenous tumor-specific T-cell and NKs or enhance the co-stimulatory activity of DCs. These studies provide the underlying mechanism of augmented activated CD8^+^ T cells and reinforced tumor-specific T-cell response in our results (Figs. 7 and 8B). Chen et al. conducted a phase I/II trial to investigate the tumor-control efficacy of DC-CIK (CIK cell product co-cultured with ex vivo-expanded DC) and pembrolizumab combination in advanced solid tumor patients [34, 35]. In this study, 64.5% and 22.5% of patients experienced disease control and tumor shrinkage, respectively [35]. According to the mechanism described above, combining DC-CIK with anti-PD-L1 antibodies (such as atezolizumab or avelumab) rather than pembrolizumab may have better efficacy in tumor control.

Kalinski et al. reported that NK aided DC anti-cancer responses [36, 37]. Similar effects were observed in another study, which utilized NKs mediated type-1 polarization of DCs to enhance tumor-specific cytotoxic lymphocyte effects in melanoma [38]. Researchers have concluded that NK is the key to DC-based immunotherapy and vaccines [39]. These findings provided a sound rationale for the present study. In our mouse model, treatment of NKDC or α-mPD-L1 partially controlled tumor growth. NKDC/α-mPD-L1 combination reduced tumor growth stronger than applying NKDC or α-mPD-L1 alone, even at a low cell dose. This result confirmed that tumor control is based on the further initiation of the cytotoxic T-cell activation cascade, not simply the NK innate immunity-killing effect. This additively tumor-control efficacy was demonstrated clearly in the cytotoxic T-lymphocyte response assay.

## Conclusion

In conclusion, our study provided an efficient and practical approach to augment the efficacy of α-mPD-L1 through co-utilizing with NKDCs. NKDC/α-mPD-L1 combination facilitates the mobilization of T lymphocytes and DCs to the tumor-bedding site and enhances tumor-specific T-cell response. Accumulated tumor-specific T cells can archive sustained tumor control. Our findings hold great potential to improve responses to current cancer immunotherapies by turning cold tumors into the hot tumor. In the future, we would continuously investigate the long-term memory and long-term tumor-control activity of NKDC/ICI combination in vivo. Additionally, we plan to confirm the proposed conception in this study via early clinical trials shortly.

### Electronic supplementary material

Below is the link to the electronic supplementary material.

**Supplementary Material 1: Supplementary Table 1.** Reagents applied in this study. **Supplementary Table 2.** Antibodies applied in this study. **Supplementary Figure 1.** Schematic illustration of NKDC gating strategy. **Supplementary Figure 2.** Schematic illustration of gating strategy of NKs and DCs. **Supplementary Figure 3.** Schematic illustration of gating strategy of regulatory cells. **Supplementary Figure 4.** Schematic illustration of gating strategy of activated CD8 T cells

## Data Availability

The raw data are available from corresponding author YC on request.
