# Supplementary material for: Enhanced tumor control activities of anti-mPD-L1 antibody and antigen-presenting cell-like natural killer cell in an allograft model
Source: BMC Cancer. 2024 Jan 26;24:136. doi: 10.1186/s12885-024-11889-4 (PMC10811836; doi:10.1186/s12885-024-11889-4)
Supplement: Supplementary file 1 — Supplementary Material 1: Supplementary Table 1. Reagents applied in this study. Supplementary Table 2. Antibodies applied in this study. Supplementary Figure 1. Schematic illustration of NKDC gating strategy. Supplementary Figure 2. Schematic illustration of gating strategy of NKs and DCs. Supplementary Figure 3. Schematic illustration of gating strategy of regulatory cells. Supplementary Figure 4. Schematic illustration of gating strategy of activated CD8 T cells [file 12885_2024_11889_MOESM1_ESM.docx]

**Supplementary Table 1. Reagents applied in this study**

| **Reagents** | **Abbreviation** | **Manufacture** |
| --- | --- | --- |
| ***For cell line maintainance*** | | |
| 2-Mercaptoethanol | 2-ME | Thermo-Fisher |
| Fetal bovine serum | FBS | Thermo-Fisher |
| Geneticin | G418 | Thermo-Fisher |
| Penicillin/Streptomycin | P/S | Thermo-Fisher |
| Rosewell Park Memorial Institute medium 1640 | RPMI-1640 | Thermo-Fisher |
| ***For NK-DC cultivation*** | | |
| Murine granulocyte monocyte-colony stimulating factor | rmGM-CSF | Thermo-Fisher |
| Murine interleukin-15 | rmIL-15 | Thermo-Fisher |
| Red blood cell lysis buffer | RBC lysis buffer | Thermo-Fisher |
| ***For activity assay*** | | |
| CellTrace^TM^ CFSE cell proliferation kit | CFSE | Thermo-Fisher |
| Cytofix/Cytoperm fixation/permeabilization kit with Golgi stop | Cytofix | BD Biosciences |
| eBioscience^TM^ Cell Stimulation Cocktail | Cell Stimulation Cocktail | Thermo-Fisher |
| Ovalbumin_257-264_ peptide (sequence: SIINFEKL) | OVA peptide | Genescript |
| PanToxilux cytotoxicity kit |  | OncoImmunin Inc. |
| ***For immunostaining and phenotype assay*** | | |
| Cell staining buffer |  | Biolegend |

Abbreviation: NKDC, antigen-presenting-cell-like natural killer cell

**Supplementary Table 2. Antibodies applied in this study**

| **Target** | **Clone** | **Fluorophore** | **Manufacture** |
| --- | --- | --- | --- |
| B220 | RA3-6B2 | APC | Biolegend |
| CD4 | GK1.5 | PE | Biolegend |
| CD8 | 53-6.7 | PE/Cy7 | Biolegend |
| CD11b* | M1/70 | PerCP/Cy5.5 | Biolegend |
| CD11c | N418 | APC/Cy7 | Biolegend |
| Fc blocker | 2.4G2 |  | BD |
| CD19 | 6D5 | PE/Cy7 | Biolegend |
| CD25 | PC61 | AF700 | BD |
| CD69* | H1.2F3 | PerCP/Cy5.5 | Biolegend |
| CD274 (PD-L1) | MIH-5 | BV421 | BD |
| CD274 (PD-L1) | B7-H1 |  | BioXCell |
| CTLA-4* | UC10-4B9 | PerCP/Cy5.5 | Biolegend |
| F4/80 | BM8 | PE | Biolegend |
| Foxp3 | MF-14 | PB | Biolegend |
| MHCII | M5/114 | BV510 | BD |
| IFN-γ | XMG1.2 | APC | Biolegend |
| Ly-6C | AL-21 | APC | Biolegend |
| Ly-6G | 1A8 | PE/Cy7 | BD |
| NK-1.1 | PK136 | BV421 | BD |
| NKp46 | 29A1.4 | PerCP/Cy5 | Biolegend |
| TCR-β | H57-597 | FITC | Biolegend |
| ***Isotype control*** | | | |
| Armenian Hamster IgG | HTK888 | PerCP/Cy5.5 | Biolegend |
| Mouse IgG2aκ | MOPC-173 |  | Biolegend |
| Rat IgG2aκ | RTK2758 | APC | Biolegend |
| Rat IgG2bκ | RTK4530 | BV421 | Biolegend |

* All antibodies enlisted above were rat anti-mouse antibodies except those labeled with *. Abbreviation: APC, allophycocyanin; A700, Alexa Fluor^®^ 700; BV421, brilliant bright 421; Cy5.5, cyanine 5.5; FITC, fluorescein isothiocyanate; PB, pacific blue; PE, phycoerythrin; PerCP, Peridinin-Chlorophyll-protein.

**
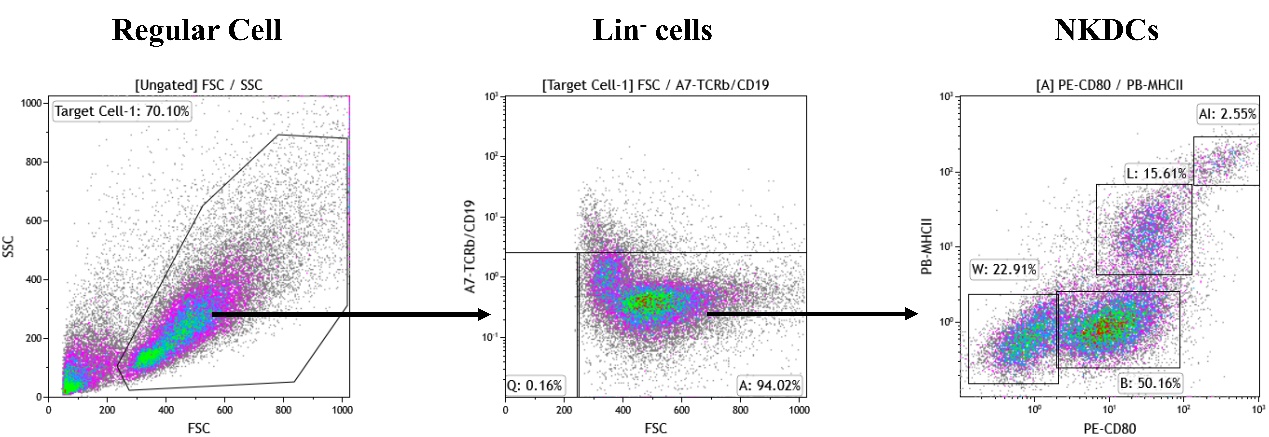
**

**Supplementary Figure 1. Schematic illustration of NKDC gating strategy.**

Cultured cells were firstly gated with FSC and SSC to route cell debris and aggregation followed by gating with TCR-β^-^CD19^-^ to remove lineage cells. Finally, NKDCs were identified via evaluation of CD80 and MHCII.

**
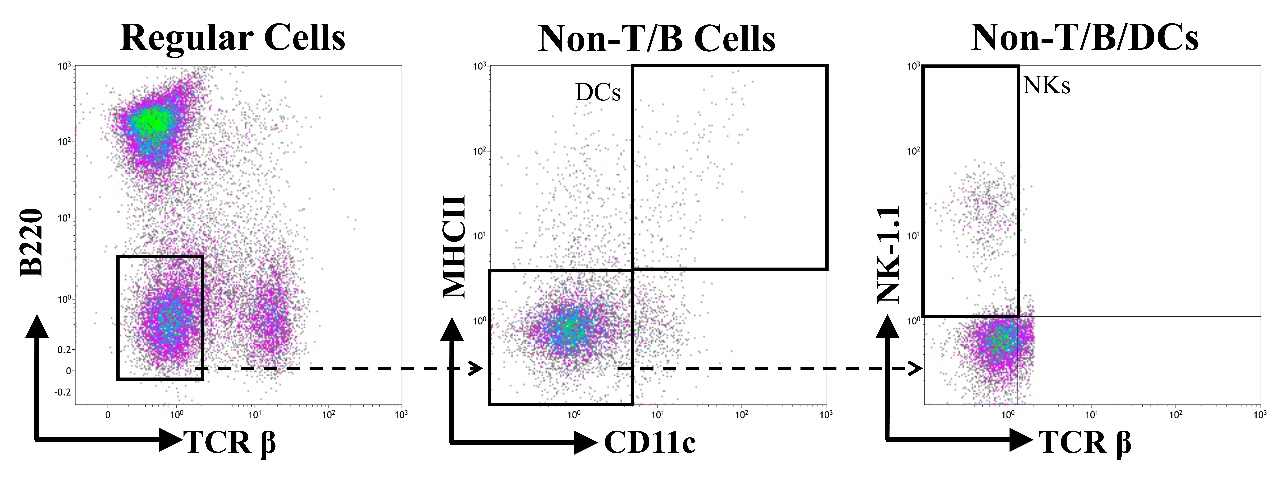
**

**Supplementary Figure 2. Schematic illustration of gating strategy of NKs and DCs.**

B220^-^TCRβ^-^ cells were firstly gated. DCs were CD11c^+^MHCII^+^ cells in B220^-^TCRβ^-^ cells, and NKs were NK-1.1^+^ cells in CD11c^-^MHCII^-^ cells.

**
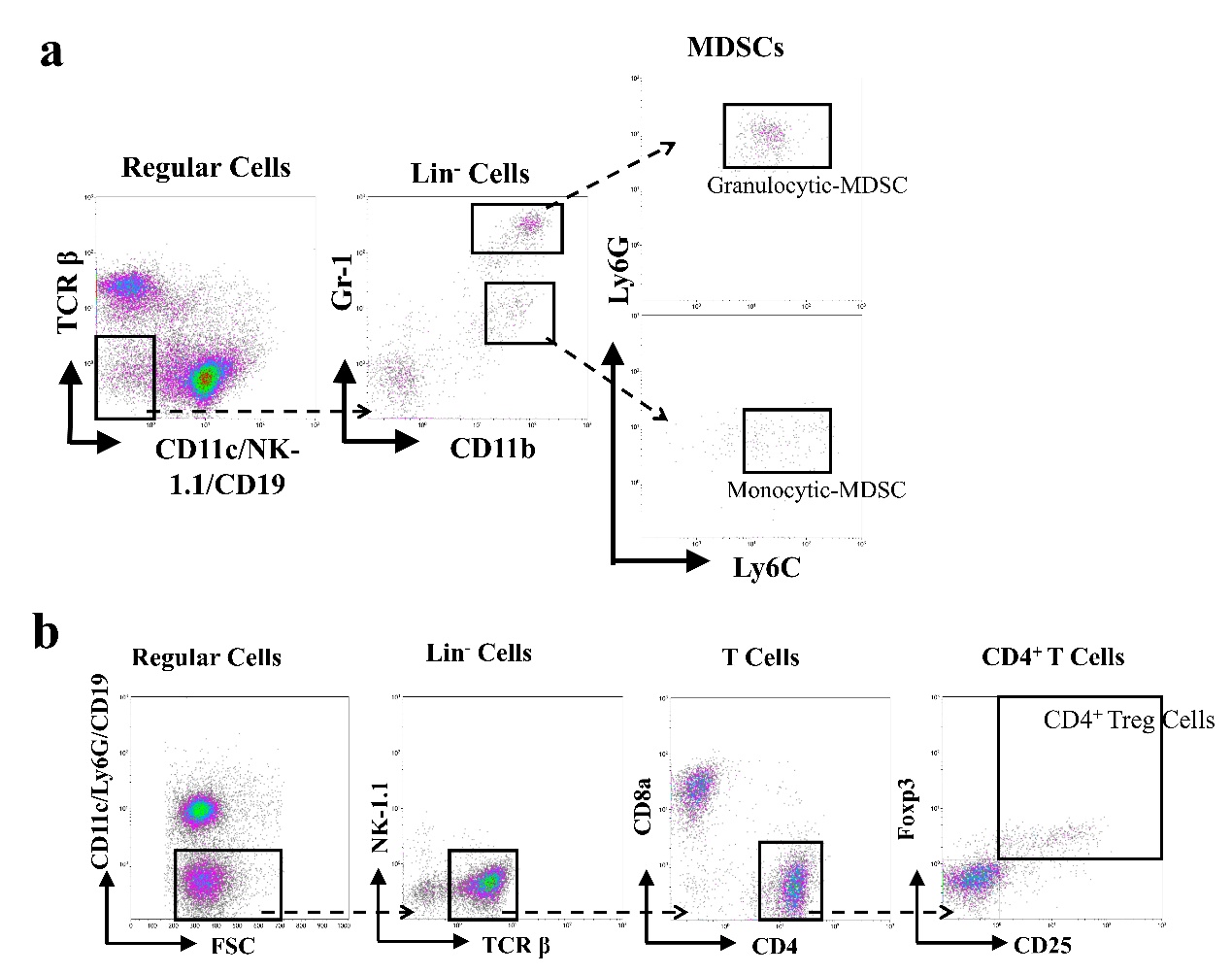
**

**Supplementary Figure 3. Schematic illustration of gating strategy of regulatory cells.**

**(a)** For granulocytic or monocytic myeloid-derived suppressor cell (MDSC) determination, the regular and lineage-negative cells, i.e., non-T/B/NK/DCs, were first gated. After that, MDSCs were determined as CD11b^+^Gr-1^+^/Gr-1^++^. G-MDSCs were determined as CD11b^+^Gr-1^++^Ly6C^++^Ly6G^+^, while M-MDSCs were determined as CD11b^+^Gr-1^+^Ly6C^+^Ly6G^+^. **(b)** For CD4^+^ Treg, non-B/ NK/granulocyte/DC regular cells were first gated. CD4^+^ T cells were subsequently selected for Foxp3/CD25 expression analysis. CD4^+^ Treg cells were determined as Foxp3^+^CD25^+^.

**
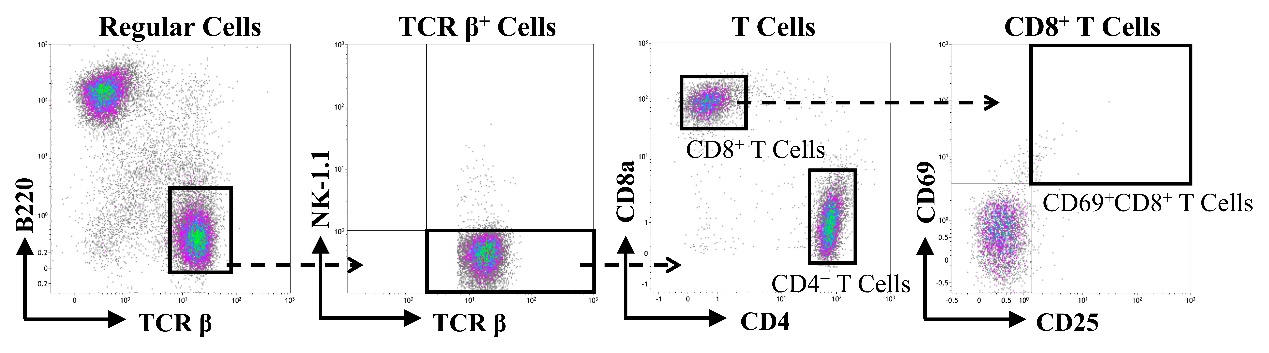
Supplementary Figure 4. Schematic illustration of gating strategy of activated CD8 T cells.**

The PBMC, sentinel and tumor infiltrating lymphocytes C57BL/6 recipients were prepared and applied to FACS analysis. To determined effector T cells, regular and NK-1.1^-^TCR β^+^ were first gated and then were separated into CD4^+^ and CD8^+^ T cells. CD8^+^ T cells were further observed with CD69 and CD25 expression level. CD8^+^ T cells with CD69^+^ were considered as activated condition.
